# The prognostic roles of circulating ALDH1^+^ tumor cell in the patients with non-small cell lung cancer

**DOI:** 10.1042/BSR20180914

**Published:** 2018-10-31

**Authors:** Shuang Tian, Ya-Nan Xing, Pu Xia

**Affiliations:** 1 Department of Cell Biology, College of Basic Medical Science, Liaoning Medical University, Jinzhou, Liaoning, P.R. China; 2 Department of Surgical Oncology, First Affiliated Hospital, China Medical University, Shenyang, Liaoning, P.R. China; 3 Department of Cell Biology, Biological Anthropology Institute, Liaoning Medical University, Jinzhou, Liaoning, P.R. China

**Keywords:** ALDH1, cancer stem cell, metastasis, NSCLC, prognosis

## Abstract

Circulating tumor cells can provide important diagnostic and prognostic information of the patients with non-small cell lung cancer (NSCLC). Aldehyde dehydrogenase 1 (ALDH1), a cancer stem cell marker, has been used in various tumors, including NSCLC. In the present study, we isolated the circulating ALDH1^+^ tumor cells from the NSCLC patients using ALDH1 as a potential marker. Higher percentage of ALDH1^+^ tumor cells was identified in blood samples from the NSCLC patients compared with normal controls. ALDH1^+^ cells were correlated with the poor prognosis of these patients by using Kaplan–Meier analysis. In the last, the tumorigenic properties of ALDH1^+^ tumor cells were determined *in vitro* and *in vivo* by using sphere assay and xenograft tumor mouse models. Our *in vitro* and *in vivo* experiments demonstrated that ALDH1 could drive the stemness of circulating NSCLC cells. Circulating ALDH1^+^ cells could be used as a prognostic marker for NSCLC.

## Introduction

Lung cancer accounts for approximately 18% of all cancer deaths worldwide [1]. Most newly diagnosed lung cancers (75–85%) are non-small cell lung cancer (NSCLC) [1]. Despite the advances in early detection, the survival rates of the NSCLC patients are still very poor [2]. Therefore, it is of great significance to find a reliable predictor for understanding the pathogenesis of NSCLC.

Asworth [3] first discovered the circulating tumor cells (CTCs) in 1869. Since then, isolation and characterization of CTCs in cancer patients became a hot topic [4]. CTCs included valuable information about tumor invasiveness, recurrence, and drug resistance [5]. Aldehyde dehydrogenase 1 (ALDH1) is a detoxifying enzyme that oxidizes aldehyde into carboxylic acid and converts retinol into retinoic acid [6]. It has been found in various tumors, including NSCLC, breast cancer, and gastric cancer [7–9]. However, to our knowledge, no previous studies showed the prognostic and biological roles of the circulating ALDH1^+^ tumor cells in the NSCLC patients.

In the present study, we isolated the circulating ALDH1^+^ tumor cells from the serum of NSCLC patients and evaluated the clinicopathological characteristics of these patients. In addition, we compared the relationship between serum soluble TNF-related apoptosis-inducing ligand (TRAIL) and circulating ALDH1^+^ tumor cells. We hope to confirm two related markers for NSCLC to increase diagnostic accuracy.

## Materials and methods

### NSCLC patient blood samples

Blood samples were obtained from 48 NSCLC patients at the First Hospital of China Medical University (Jan 2007 to Dec 2010). Blood samples from 16 healthy individuals were used as control. The clinical investigation was conducted according to the principles expressed in the Helsinki Declaration of 1975. The Liaoning Medical University Ethics Committee reviewed and approved the protocol of the present study, and a written informed consent was required from the participants.

### ELISA

Blood samples were centrifuged at 2500×***g*** for 30 min. For the measurement of serum TRAIL (sTRAIL), analyses were performed by using an ELISA kit (R&D Systems, Minneapolis, MN, U.S.A.) in accordance with the manufacturer’s instructions, and analyzed with an ELISA reader at 450 nm.

### Circulating ALDH1^+^ tumor cells sorting

Each blood sample (7.5 ml) was mixed with the magnetic bead-labeled anti-human EpCAM monoclonal antibody. Then, cells were added to the magnetic separation column and captured by using the magnetic field. Isolated cells were stained with FITC-conjugated anti-cytokeratin (CK) and PE-conjugated anti-CD45 (StemCell Technologies, Miami, FL, U.S.A.) for 1 h, stained with DAPI for 20 min. As the instruction of the manufacturer, Aldehyde Dehydrogenase-Based Cell Detection Kit (StemCell Technologies) was used to determine ALDH1 enzymatic activity in isolated circulating tumor cells. Briefly, cells (1 × 10^6^/ml) were suspended in ALDEFLUOR Assay Buffer. ALDEFLUOR Reagent BODIPY™ (1.25 μl) was added as a substrate to measure ALDH1 enzymatic activity in cells. ALDEFLUOR/DEAB treated cells were used to define negative gates.

### Sphere assay

As our previous method, cells (6 × 10^4^ cells/well) were plated in six-well, ultra-low attachment plates under serum-free, sphere-specific conditions [10]. After culture for 7 days, spheres were fixed in 4% paraformaldehyde (Sigma Chemicals, St Louis, MO, U.S.A.), stained with crystal violet (Beyotime, Shanghai, China), and visible under a light microscope (Olympus CX31, Olympus, Tokyo, Japan).

### Transwell assay

The migration assay was performed by using the Boyden chamber (8 μM pore size polycarbonate membrane; Cell Biolabs, San Diego, CA, U.S.A.). Briefly, the upper chamber was loaded with 100 μl of cell suspension (3 × 10^5^ cells/ml) and the lower chamber was loaded with 600 μl of DMEM containing 10% FBS. After incubation for 12 h, the filter was fixed in 4% paraformaldehyde (Sigma Chemicals) and stained with crystal violet (Beyotime). The cells on the upper side of the filter were wiped off using a cotton swab. The cells that migrated to the undersurface of the membrane were counted using a light microscope (Olympus CX31).

### *In vivo* tumor study

All animal experiments were performed using protocols approved by Liaoning Medical University Animal Care and Use Committee. All experimental procedures were carried out in strict accordance with the Guidelines for Laboratory Animal Welfare Ethics Review. As our previous method [10], unsorted, ALDH1^−^, or ALDH1^+^ cells (5 × 10^6^/100 μl) were subcutaneously injected into male BALB/c mice (5- to 7-week-old, 17–20 g; Charles River, Wilmington, MA, U.S.A.). All mice were housed and maintained under specific pathogen-free conditions. Every 5 days until the end of the experiment, tumors were excised, formalin-fixed, and paraffin-embedded. For each tumor, measurements were made using calipers, and tumor volumes were calculated as follows: length × width^2^ × 0.52 [11]. Paraffin-embedded tissues were cut into sections with a thickness of 4 μm. ALDH1 antibody or Ki67 antibody (Santa Cruz Biotechnology, Santa Cruz, CA, U.S.A.) was incubated with sections at 1:200 overnight at 4°C.

### Statistical analysis

All statistical analyses were carried out by using GraphPad Prism 5 software (GraphPad Software, San Diego, CA, U.S.A.). Statistical analysis was performed using a two-tailed un-paired Student’s *t*-test. Discrete variables were compared by the Chi-squared test. Survival curves were plotted using the Kaplan–Meier method. *P* values <0.05 were considered to indicate statistically significant differences. All quantitative data presented are the mean ± SEM.

## Results

### The levels of serum TRAIL in the NSCLC patients

**Figure 1:**
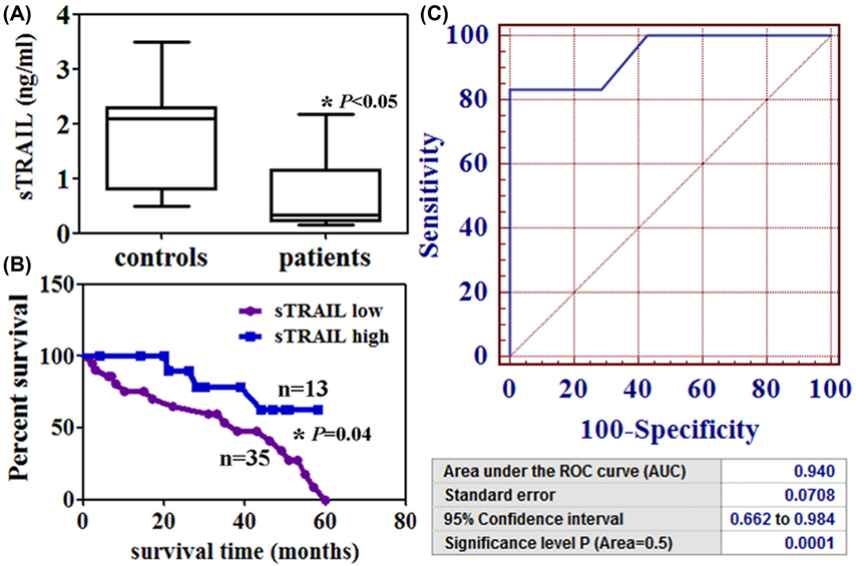
TRAIL from the serum of NSCLC patients. (**A**) sTRAIL levels were detected by using ELISA. *P<0.05 (**B**) Kaplan–Meier curves of the cumulative survival rate of the NSCLC patients based on their sTRAIL levels. *P=0.04 (**C**) ROC curve analysis for sTRAIL diagnosis in these patients.

The concentration of sTRAIL in 48 patients ranged from 0.15 to 2.17 ng/ml with a median of 0.68 ng/ml (Figure 1A). The sTRAIL levels were lower in the patients than that in healthy controls (*P*<0.05, Figure 1A). Low sTRAIL level (<0.68 ng/ml) was associated with a shorter survival time of the NSCLC patients (*P*<0.05, Figure 1B). The ROC curves of sTRAIL revealed a strong discrimination between the NSCLC patients with an AUC of 0.940 (*P*<0.05, Figure 1C). Table 1 showed the relationship between sTRAIL levels and clinicopathologic findings. sTRAIL levels were associated with differentiation (*P*=0.001), lymphatic invasion (*P*=0.001), venous invasion (*P*=0. 0.006), and pN category (*P*=0.016).

**Table 1:** Relationship between sTRAIL or circulating ALDH1^+^ tumor cells and clinicopathological parameters of patients with NSCLC

Clinicopathological features	sTRAIL					ALDH1^+^			
	*n*	low	high	χ^2^	*P*	-/low	High	χ^2^	*P*
Sex				0.40	0.527			1.11	0.292
Female	10	6	4			5	5		
Male	38	29	9			28	10		
Age (years)				0.13	0.716			1.34	0.247
<55	7	5	2			3	4		
≥55	41	30	11			30	11		
Differentiation				12.2	**0.001**			9.06	**0.003**
Well or moderate	11	3	8			3	8		
Poor	37	32	5			30	7		
Lymphatic invasion				7.24	**0.001**			10.9	**0.001**
-	20	10	10			8	12		
+	28	25	3			25	3		
Venous invasion				7.71	**0.006**			0.04	0.846
-	25	23	2			17	8		
+	23	12	11			16	7		
Histological type				0.88	0.644			3.81	0.149
Squamous cell	15	11	4			12	3		
Adenocarcinoma	14	9	5			11	3		
Large cell	19	15	4			10	9		
Tumor size				0.08	0.771			2.79	0.095
<3 cm	8	6	2			3	5		
≥3 cm	40	29	11			30	10		
pN category				10.4	**0.016**			4.41	0.220
pN0	10	5	5			6	4		
pN1	8	6	2			4	4		
pN2	14	8	6			9	5		
pN3	16	16	0			14	2		

Abbreviation: χ^2^, Chi-square distribution.

Bold terms mean the values have statistical significance (<0.05).

### Circulating ALDH1^+^cells from the serum of NSCLC patients

**Figure 2:**
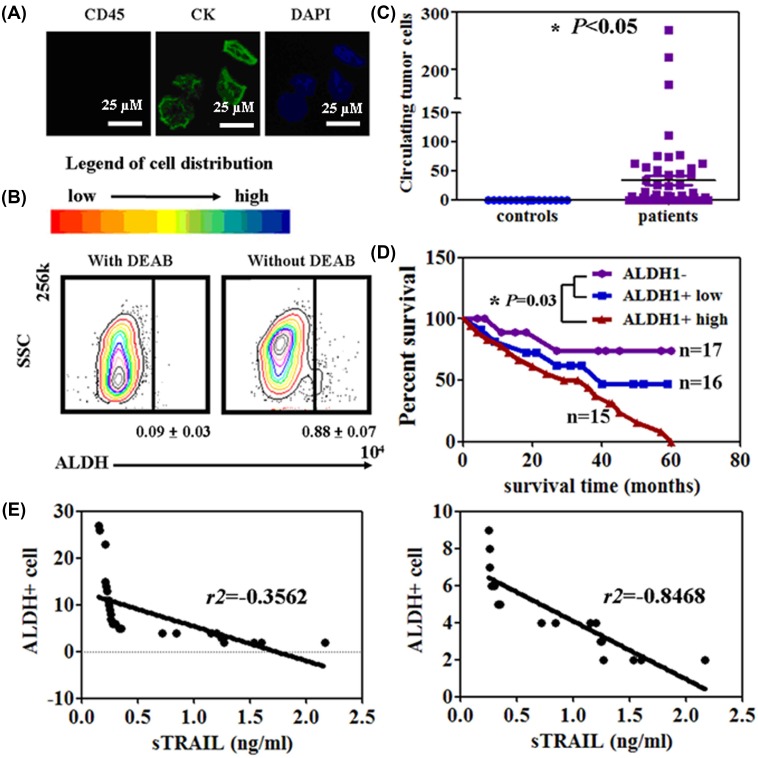
Isolation of circulating ALDH1^+^ cells from the serum of NSCLC patients. (**A**) CTCs are identified by using the Immunofluorescence assay with CK, DAPI, and CD45. (**B**) ALDEFLUOR FACS analysis of the CTCs. (**C**) Circulating ALDH1^+^ cells were calculated by using FCM in patients and healthy controls. *P<0.05 (**D**) Kaplan–Meier curves of the cumulative survival rate of the NSCLC patients based on the number of circulating ALDH1^+^ cells. *P=0.03 (**E**) Correlation between sTRAIL and circulating ALDH1^+^ cells in the NSCLC patients.

Immunofluorescence assays were performed in isolated circulating tumor cells. The isolated cells showed CK expression, but not with CD45 expression (Figure 2A). About 0.79 ± 0.04% of the total circulating tumor cells showed high ALDH1 activity (Figure 2B). Compared with healthy controls, the NSCLC patients showed a higher number of ALDH1^+^ cells (0–268, mean = 33.5) (*P*<0.05, Figure 2C). The median overall survival was significantly shorter in the group with the high level ALDH1^+^ patients (ALDH1^+^ cell number >10) compared with the ALDH1^−^ patients and low level ALDH1^+^ patients (ALDH1^+^ cell number <10) (*P*<0.05, Figure 2D). The results of the association of ALDH1^+^ cells with the clinicopathological characteristics of the sampled patients were summarized in Table 1. ALDH1^+^ cells were associated with tumor differentiation (*P*=0.003) and lymphatic invasion (*P*=0.001).

Furthermore, the sTRAIL level was significantly negatively correlated to the number of ALDH1^+^ cells in the high level ALDH1^+^ patients (*r*^2^ = –0.8468, *P*<0.05, Figure 2E). However, no association was observed between sTRAIL levels and ALDH1^+^ cells in the low level ALDH1^+^ patients (*r*^2^ = –0.3562, *P*>0.05, Figure 2E).

### Tumorigenic properties of ALDH1^+^ cells both *in vitro* and *in vivo*

**Figure 3:**
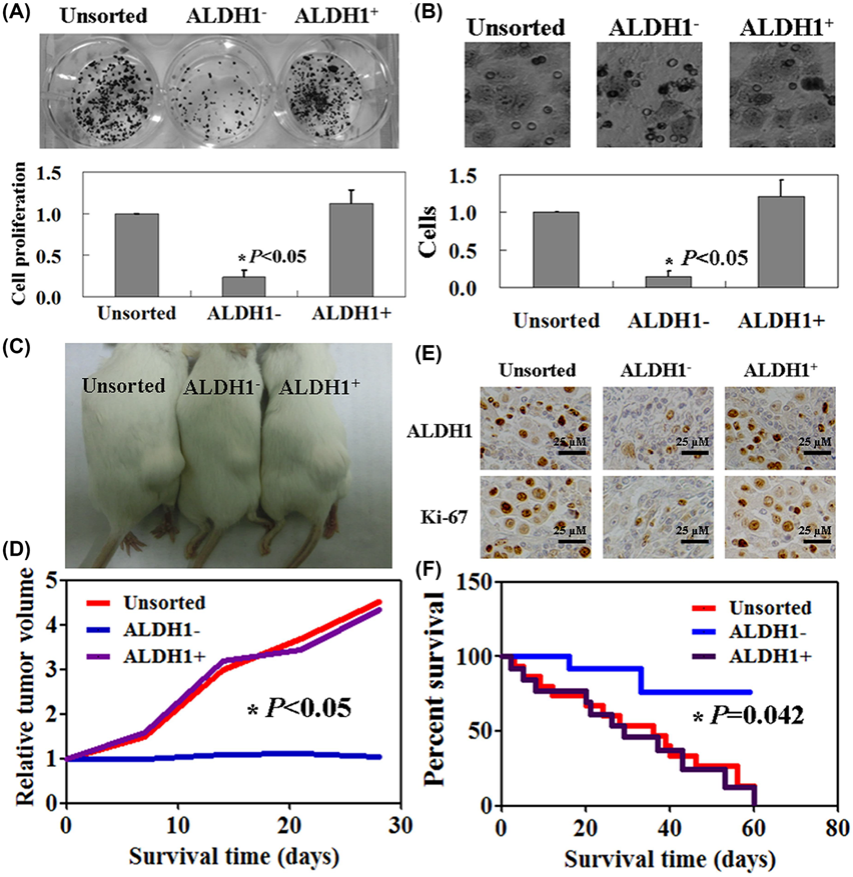
Tumorigenic properties of ALDH1^+^ cells *in vitro* and *in vivo* (**A**) The proliferation rate of unsorted, ALDH1^−^, and ALDH1^+^ cells was assayed by using the colony formation assay. *P<0.05 (**B**) Transwell assay was used to detect the mobility of unsorted, ALDH1^−^, and ALDH1^+^ cells. *P<0.05 (**C**) Macroscopic appearance of subcutaneous tumors in unsorted, ALDH1^−^, and ALDH1^+^ cells injected mouse models. (**D**) The tumor volume of each group as described above. *P<0.05 (**E**) Immunohistochemical staining of resected tumor tissues from each group using ALDH1 and Ki67. (**F**) Kaplan–Meier survival curves of each group as described above. *P<0.05

Colony formation assay was performed to detect the proliferation of unsorted, ALDH1^−^, and ALDH1^+^ cells. Both unsorted and ALDH1^+^ cells showed a higher proliferation ratio than ALDH1^−^ ones (*P*<0.05, Figure 3A). Transwell assay showed the mobility of unsorted, ALDH1^−^, and ALDH1^+^ cells. Compared with unsorted cells, there was a significantly decreased migration of ALDH1^−^ cells (*P*<0.05), but no significantly changed migration of ALDH1^+^ ones (*P*>0.05, Figure 3B)

The similar results were observed *in vivo*. Unsorted, ALDH1^−^, and ALDH1^+^ cells had the distinct abilities to form the tumor into immunocompromised mice (Figure 3C). The tumor volume of mice in unsorted and ALDH1^+^ group was 1.5–4.4 times than ALDH1^−^ group (*P*<0.05, Figure 3D). The tumor volume of ALDH1^−^ mice showed no significant changes through the beginning to the end of the experiment. High Ki-67 expression was found in the tissues of mice in unsorted and ALDH1^+^ group (Figure 3E). The survival rate of ALDH1^+^ cells and unsorted cells injected mice was significantly lower than the mice in ALDH1^−^ group (*P*<0.05, Figure 3F).

## Discussion

Previous studies have confirmed CTC identification can provide prognostic and predictive information of NSCLC [12–15]. Early detection and prognostic assessment are needed in order to improve the therapeutic intervention for NSCLC. So, we carried out the present study in order to find a more accurate marker for NSCLC. As noted in the ‘Introduction’ section, ALDH1 is a widely used cancer stem cell marker. To our knowledge, no previous studies reported the prognostic roles of the circulating ALDH1^+^ cells in cancer patients. In the present study, we isolated the circulating ALDH1^+^ cells and confirmed the cells could be used as a prognostic marker for NSCLC patients. In addition, ALDH1^+^ cells were associated with tumor differentiation and lymphatic invasion. These results indicated that high ALDH1 expression could induce cancer cell migration. By culturing ALDH1^+^ cells, we further found that ALDH1^+^ cells have higher proliferation and migration rates *in vitro*. The similar results were confirmed by using immunocompromised mice. In the study of Pan et al. [16], they found that ALDH1 positive invasive breast cancers were significantly with high Ki67 expression. A significant positive correlation was found between ALDH1 and Ki67 in astrocytic gliomas [17]. Consistent with previous studies, we also found the positive relationship of ALDH1 and ki67 in established xenograft tumors.

Another main finding of the present study is that sTRAIL may be a useful marker for detecting patients with NSCLC. TRAIL is a type 2 transmembrane death ligand that causes apoptosis in various tumors and cancer cells, including NSCLC cells [18]. In our previous study, we confirmed that the cytotoxicity of TRAIL was confirmed in H460 cells and *in vivo* [18]. Based on the data of the present study, we found that the sTRAIL level was negatively correlated to the number of circulating ALDH1^+^ cells in the patients with NSCLC.

## Conclusion

There are three main conclusions in the present study: (1) circulating ALDH1^+^ cells could be used as a prognostic marker for NSCLC; (2) ALDH1^+^ cells were associated with tumor differentiation and lymphatic invasion; (3) negative relationship between sTRAIL and ALDH1^+^ cells. However, the potential mechanism should be detected in future studies.

## Abbreviations

ALDH1: aldehyde dehydrogenase 1
CTC: circulating tumor cell
NSCLC: non-small cell lung cancer
sTRAIL: serum TRAIL
TNF: tumor necrosis factor
TRAIL: TNF-related apoptosis-inducing ligand
